# Extraskeletal Myxoid Chondrosarcoma Mimicking Myoepithelial Tumor

**DOI:** 10.1155/crip/2779791

**Published:** 2026-05-03

**Authors:** Bowen Jiang, Masatake Matsuoka, Kanako C. Hatanaka, Shinichi Nakazato, Harumi Nakamura, Seiji Nakamura, Hirokazu Shimizu, Tomohiro Onodera, Shinya Tanaka, Yutaka Hatanaka, Norimasa Iwasaki

**Affiliations:** ^1^ Department of Orthopaedic Surgery, Faculty of Medicine and Graduate School of Medicine, Hokkaido University, Sapporo, Hokkaido, Japan, hokudai.ac.jp; ^2^ Center for Development of Advanced Diagnostics, Hokkaido University Hospital, Sapporo, Japan, hokudai.ac.jp; ^3^ Department of Surgical Pathology, Hokkaido University Hospital, Sapporo, Hokkaido, Japan, hokudai.ac.jp; ^4^ Laboratory of Genomic Pathology, Osaka International Cancer Institute, Osaka, Japan, pref.osaka.jp; ^5^ DNA Chip Research Inc, Kawasaki-shi, Kanagawa, Japan; ^6^ Department of Cancer Pathology, Faculty of Medicine, Hokkaido University, Sapporo, Hokkaido, Japan, hokudai.ac.jp

**Keywords:** extraskeletal myxoid chondrosarcoma, myoepithelial tumor, RNA panel sequencing, soft tissue sarcoma

## Abstract

Extraskeletal myxoid chondrosarcoma (EMC) is a rare soft tissue sarcoma defined by its characteristic multinodular myxoid architecture and distinctive clinicopathological features. Although EMC typically exhibits a multinodular myxoid architecture, its histologic variability and frequent immunophenotypic overlap with other myxoid tumors can make diagnosis challenging. We report a case of EMC arising around the left knee of a 73‐year‐old male patient. Histologically, the tumor exhibited abundant myxoid stroma with lace‐like and haphazard cellular arrangements and focal epithelioid morphology. Immunohistochemically, the lesion showed diffuse positivity for several myoepithelial markers, including epithelial membrane antigen, *α*–smooth muscle actin, HHF35, calponin, and p63. However, the absence of cytokeratin and SOX10 expression raised diagnostic uncertainty despite the myoepithelial‐like immunoprofile. Because immunohistochemistry remained inconclusive, targeted RNA sequencing was performed on formalin‐fixed, paraffin‐embedded tissue. A *TAF15*::*NR4A3* fusion transcript was identified, leading to revision of the initial diagnosis to EMC. At 3‐year follow‐up, the patient remains free of recurrence or metastasis. This case demonstrates the potential for EMC to mimic myoepithelial tumors and supports the use of molecular analysis when histologic or immunohistochemical findings are insufficient for diagnosis.

## 1. Introduction

Extraskeletal myxoid chondrosarcoma (EMC) is a rare soft tissue sarcoma that typically arises in the deep soft tissues of the extremities of middle‐aged to elderly adults [1, 2]. Histologically, EMC is characterized by a multinodular architecture, abundant myxoid stroma, and uniform spindle to epithelioid cells arranged in strands and cords [3]. Although originally described as a distinct cartilaginous tumor, subsequent studies have demonstrated that EMC lacks true chondroid differentiation and is instead defined by its characteristic molecular alterations involving *NR4A3* fusion genes [4, 5]. These molecular events, most commonly *EWSR1*::*NR4A3* and less frequently *TAF15*::*NR4A3*, are regarded as highly characteristic of EMC and form an essential component of contemporary diagnostic criteria.

Despite its distinctive molecular signature, EMC remains diagnostically challenging because its morphology overlaps with a broad spectrum of myxoid soft tissue tumors [6]. Immunohistochemistry is frequently of limited value in narrowing the differential diagnosis, as EMC often shows nonspecific or variable expression of epithelial or myoepithelial markers. A recent report [7] has highlighted that a subset of EMCs exhibit keratin or myoepithelial marker positivity, further complicating the diagnostic process and creating additional challenges.

Herein, we describe a unique case of EMC of the left knee joint that was initially considered to be a myoepithelial tumor due to diffuse staining for multiple myoepithelial markers. Molecular analysis ultimately revealed a *TAF15*::*NR4A3* fusion, leading to a revision of the initial diagnosis from myoepithelial tumor to EMC. This case underscores the diagnostic challenges associated with EMCs exhibiting myoepithelial immunophenotypes and highlights the critical role of molecular testing in differentiating EMC from other myxoid soft tissue tumors.

## 2. Case Report

**Figure 1:**
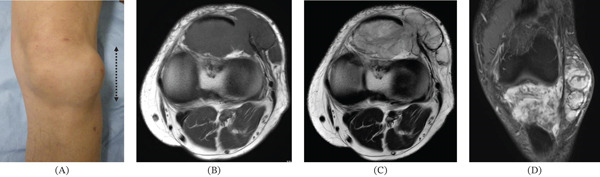
Findings at initial presentation and magnetic resonance imaging findings. (A) Findings on primary encounter. An elastic and firm tumor (black arrow: 10 cm) was evident in the left knee joint. (B–D) Magnetic resonance imaging findings. (B) Axial T1‐weighted spin echo. (C) Axial T2‐weighted spin echo. (D) T1 gadolinium‐enhanced image.

**Figure 2:**
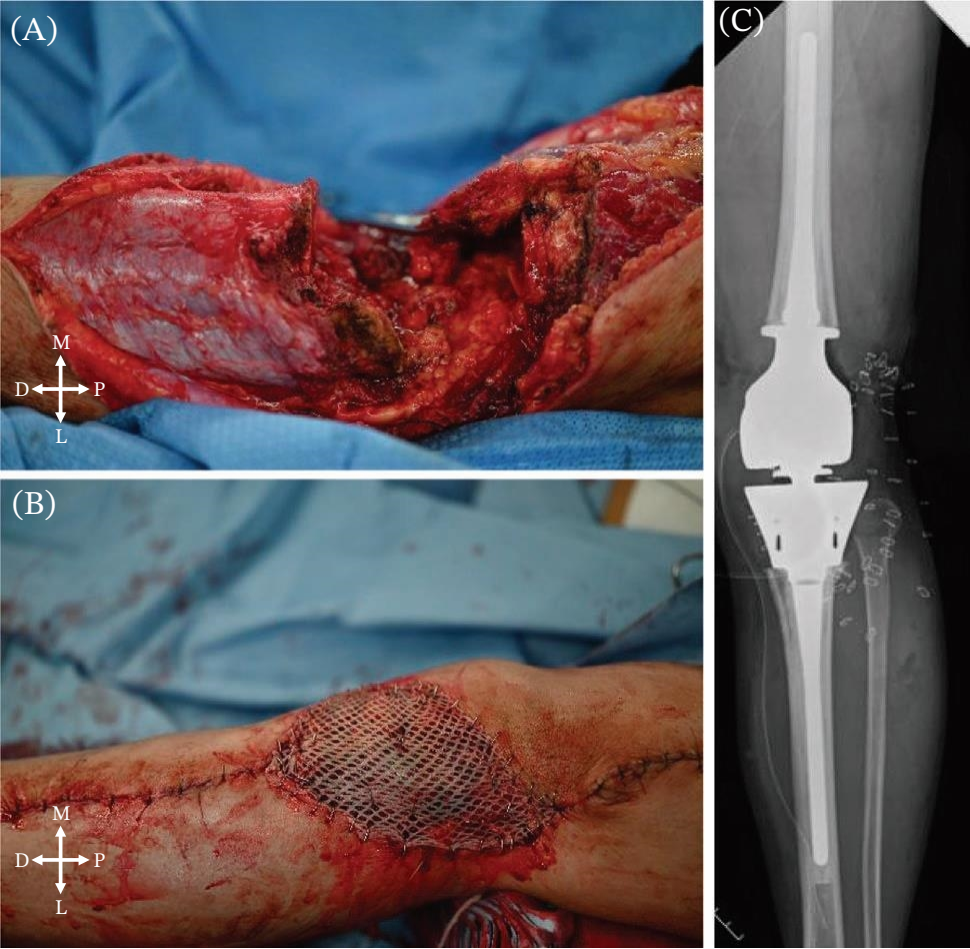
Operative procedure and postoperative radiograph. (A) An extracapsular resection including the patella was performed, followed by reconstruction using a distal femoral replacement. (B) The extensor mechanism was restored by suturing the quadriceps tendon to a gastrocnemius muscle flap including Achilles tendon, and skin grafting was carried out to complete the procedure. A postoperative radiograph is shown in (C).

A 73‐year‐old male presented with a 2‐year history of left knee swelling that had progressively enlarged over the 6 months preceding his referral, leading to an initial evaluation at a local clinic and subsequent transfer to our hospital for definitive assessment. Physical examination revealed a relatively well‐defined, deep‐seated mass (10 cm in diameter) around the left knee joint (Figure 1A). General laboratory tests were unremarkable. Magnetic resonance imaging showed a multilobulated solid tumor with high T2 signal intensity and marked contrast enhancement, showing extension from the subcutaneous tissue into the intra‐articular region (Figure 1B–D). As described subsequently, a percutaneous needle biopsy revealed a malignant soft tissue tumor with abundant myxoid stroma. Staging imaging showed no evidence of distant metastasis, and the patient was deemed a candidate for local treatment. Following an extracapsular resection including the patella, the limb was reconstructed using a distal femoral replacement (Figure 2); the extensor mechanism was restored by suturing the quadriceps tendon to a gastrocnemius muscle flap, including the Achilles tendon [8], followed by skin grafting for soft tissue coverage. No adjuvant therapy was administered postoperatively.

**Figure 3:**
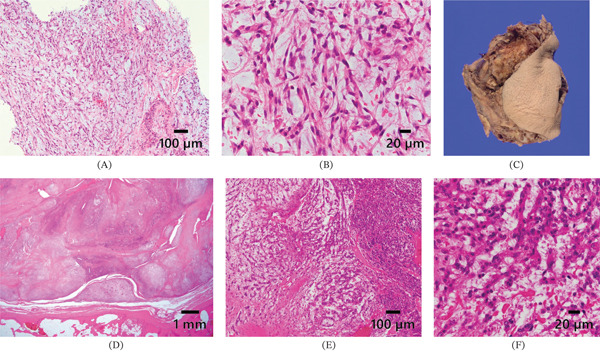
Pathological findings. (A) Histopathology of the percutaneous needle biopsy specimen showing a myxoid spindle cell neoplasm. (B) Higher magnification view of (A), showing bland spindle cells arranged in a haphazard pattern. (C) Gross appearance of the resected tumor. (D) Histopathology of the resected specimen showing a neoplasm with multinodular components. (E) Higher magnification revealing variable cellularity with areas showing cord‐like and lace‐like patterns. (F) Highest magnification demonstrating tumor cells with epithelioid morphology (A, B, D–F: hematoxylin and eosin staining; scale bars are shown in each figure).

**Figure 4:**
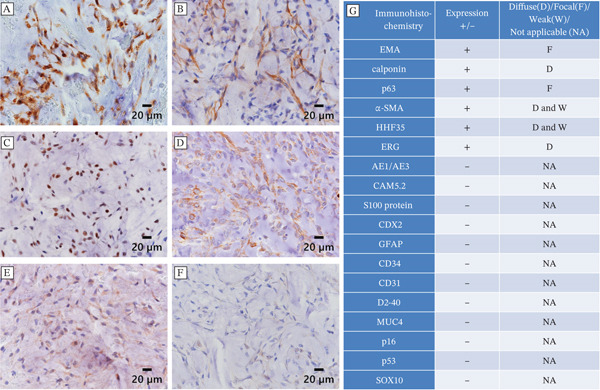
Immunohistochemical findings. (A–F) Representative immunohistochemical staining of the tumor. The tumor cells are positive for (A) EMA, (B) calponin, and (C) p63. (D) *α*SMA and (E) HHF35 show weak positivity. (F) SOX10 is negative. Scale bars: 20 *μ*m. (G) The overall results are summarized in the table.

Pathological findings from both the biopsy and the subsequent surgical specimen were largely consistent. Initially, histopathological examination of the percutaneous needle biopsy specimen demonstrated bland spindle‐shaped tumor cells arranged in a haphazard pattern within a myxoid stroma (Figure 3A,B). Following surgical excision, gross examination of the resected specimen revealed a multinodular, gelatinous tumor (Figure 3C). Subsequent histopathological evaluation of the surgical specimen showed a well‐demarcated tumor composed of multinodular components with variable cellularity (Figure 3D). At higher magnification, although some areas exhibited increased cellularity, the tumor cells were predominantly sparsely distributed within an abundant myxomatous stroma, frequently arranged in cord‐like and lace‐like networks (Figure 3E). Foci of epithelioid tumor cells, along with a small focus of tumor necrosis, were also observed (Figure 3F). Overall, the morphological features were consistent with a myxoid‐rich neoplastic lesion. Immunohistochemically, the tumor cells were positive for epithelial membrane antigen (EMA; focal) (Figure 4A), calponin (Figure 4B), p63 (focal) (Figure 4C), *α*–smooth muscle actin (*α*‐SMA; weak) (Figure 4D), muscle‐specific actin (MSA) (clone HHF35; weak) (Figure 4E), and ETS‐related gene (ERG) and showed a Ki‐67 labeling index of approximately 5%. The tumor cells were negative for a wide range of immunohistochemical markers, including cytokeratin AE1/AE3, cytokeratin CAM5.2, synaptophysin, S100 protein, caudal‐type homeobox protein 2 (CDX2), desmin, glial fibrillary acidic protein (GFAP), CD34, CD31, podoplanin (D2‐40), mucin‐4 (MUC4), cyclin‐dependent kinase inhibitor 2A (p16), p53 (wild‐type immunostaining pattern), and SRY‐box transcription factor 10 (SOX10) (Figure 4F). The overall results are summarized in Figure 4G. Although the immunophenotype suggested a myoepithelial neoplasm, the absence of cytokeratin expression and SOX10, typically positive in a majority of soft tissue myoepithelial tumors, necessitated cautious interpretation.

**Figure 5:**
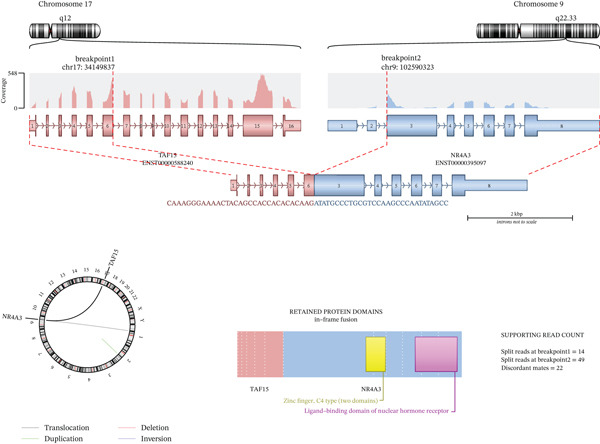
Targeted RNA sequencing analysis demonstrating a *TAF15*::*NR4A3* fusion. The fusion involved breakpoints on Chromosome 17 (*TAF15*) and Chromosome 9 (*NR4A3*), with exon–intron structures shown for both partner genes. A circos plot illustrates the fusion junction, and retained protein domain analysis demonstrates preservation of key functional regions. Supporting read counts for the fusion event are shown on the right.

To further characterize the lesion, molecular analysis was conducted as part of an institutional research framework for fusion gene detection in rare cancers. RNA extraction and targeted RNA panel sequencing were performed on formalin‐fixed, paraffin‐embedded (FFPE) tissue using a validated clinical sequencing platform. A targeted RNA fusion assay—utilizing platforms such as the TruSight RNA Pan‐Cancer Panel (Illumina) and the FusionPlex Pan Solid Tumor v2 (Invitae)—was conducted in accordance with institutional protocols and the manufacturer’s instructions. As part of this broader molecular assessment, the assay identified a *TAF15*::*NR4A3* fusion transcript, which allowed the initial diagnostic impression to be revised to EMC (Figure 5).

The postoperative course was uneventful. At the most recent follow‐up, 3 years after surgery, the patient remained free of local recurrence and distant metastasis.

## 3. Discussion

In the present case, the initial histopathological assessment suggested a myoepithelial neoplasm, as the tumor exhibited an immunophenotype with positivity for several myoepithelial markers. However, the absence of cytokeratin expression—commonly observed in soft tissue myoepithelial tumors—and the lack of SOX10 expression necessitated diagnostic caution. To further characterize the lesion, an extended molecular assessment was undertaken within our institutional research framework for rare soft tissue sarcomas. A targeted RNA fusion panel subsequently identified a *TAF15*::*NR4A3* fusion transcript, leading to revision of the initial diagnostic impression to EMC. This case therefore illustrates the diagnostic challenges that arise when EMC displays overlapping immunophenotypic features with myoepithelial tumors.

EMC is defined by rearrangements involving *NR4A3*, and molecular confirmation has become an increasingly important component of its diagnosis [9]. Although EMC typically displays a characteristic lobulated myxoid architecture, its histologic variability and frequent overlap with other myxoid tumors can pose significant diagnostic challenges [10]. Sugino et al. [7] reported a four‐case series of keratin‐positive EMC, emphasizing that this immunophenotypic subset may closely mimic myoepithelial tumors. In their series, two of the four tumors were initially considered most compatible with myoepithelial tumor because the combination of conspicuous stromal fibrosis and keratin expression created a misleading immunophenotypic profile, similar to the diagnostic challenge encountered in our case. These findings underscore that positivity for myoepithelial markers, particularly when accompanied by fibrotic or hyalinized stroma, can obscure the classic features of EMC and complicate histopathological interpretation.

The present case exemplifies this diagnostic challenge. On hematoxylin and eosin staining, the differential diagnosis initially included several myxoid neoplasms, such as low‐grade myxofibrosarcoma, low‐grade fibromyxoid sarcoma, and EMC, based on the myxoid‐rich morphology. Although the tumor exhibited diffuse positivity for multiple myoepithelial markers—including EMA, *α*‐SMA, HHF35, calponin, and p63—it remained negative for SOX10, a sensitive marker for Schwannian and myoepithelial differentiation. Although SOX10 negativity does not definitively exclude myoepithelial lineage, it should raise suspicion when discordant with other markers [11]. In this case, the immunophenotypic overlap between EMC and myoepithelial tumor underscored the limitations of relying solely on immunohistochemistry.

This case underscores two critical diagnostic insights: first, that EMC can manifest with extensive myoepithelial marker expression—posing significant diagnostic challenges in cases of morphological ambiguity—and second, that NR4A3 fusion testing serves as a vital diagnostic adjunct when immunohistochemistry is inconclusive. Incorporating molecular diagnostics into the standard evaluation of myxoid soft tissue tumors is essential, particularly in cases with overlapping histological or immunophenotypic features.

In conclusion, this case demonstrates that EMC can present with extensive myoepithelial marker expression, leading to significant diagnostic challenges when morphology and immunohistochemistry are inconclusive. Recognition of this atypical immunophenotype is important, and targeted *NR4A3* fusion analysis may be helpful in refining the diagnosis. Awareness of this diagnostic variability can aid pathologists in appropriately evaluating myxoid soft tissue tumors with overlapping features.

## Author Contributions

M.M. was involved in the design of the study, performed the clinical assessment and analysis and interpretation of data, and drafted and revised the manuscript. B.J., K.C.H., S.N., H.N., and S.N. were involved in the design of the study, assisted with data interpretation, and revised the manuscript for important intellectual content. H.S., T.O., S.T., Y.H., and N.I. were involved in the design of the study and data acquisition and revised the manuscript critically for important intellectual content.

## Funding

No funding was received for this manuscript.

## Disclosure

All authors have read and approved the final manuscript.

## Ethics Statement

In accordance with the regulations of our institution, ethics committee approval was not required for this case report. The patient provided written informed consent for the publication of this report and any accompanying images. This case report has been conducted in compliance with the principles of the Declaration of Helsinki of 1975.

## Consent

Written informed consent was obtained from the patient for publication of this case report and any accompanying images.

## Conflicts of Interest

The authors declare no conflicts of interest.

## Data Availability

The data that support the findings of this study are available on request from the corresponding author. The data are not publicly available due to privacy or ethical restrictions.
